# The miR-200 family in normal mammary gland development

**DOI:** 10.1186/s12861-021-00243-7

**Published:** 2021-08-28

**Authors:** Majesta J. Roth, Roger A. Moorehead

**Affiliations:** Department of Biomedical Sciences, Ontario Veterinary College, University of Guelph, Guelph, ON Canada

**Keywords:** microRNA, miR-200, Mammary development, Lactation, Cancer

## Abstract

The miR-200 family of microRNAs plays a significant role in inhibiting mammary tumor growth and progression, and its members are being investigated as therapeutic targets. Additionally, if future studies can prove that miR-200s prevent mammary tumor initiation, the microRNA family could also offer a preventative strategy. Before utilizing miR-200s in a therapeutic setting, understanding how they regulate normal mammary development is necessary. No studies investigating the role of miR-200s in embryonic ductal development could be found, and only two studies examined the impact of miR-200s on pubertal ductal morphogenesis. These studies showed that miR-200s are expressed at low levels in virgin mammary glands, and elevated expression of miR-200s have the potential to impair ductal morphogenesis. In contrast to virgin mammary glands, miR-200s are expressed at high levels in mammary glands during late pregnancy and lactation. miR-200s are also found in the milk of several mammalian species, including humans. However, the relevance of miR-200s in milk remains unclear. The increase in miR-200 expression in late pregnancy and lactation suggests a role for miR-200s in the development of alveoli and/or regulating milk production. Therefore, studies investigating the consequence of miR-200 overexpression or knockdown are needed to identify the function of miR-200s in alveolar development and lactation.

## Background

Mammals evolved mammary glands to generate milk to nourish their offspring. Milk is a complex solution containing proteins, fats, carbohydrates, vitamins and other nutrients that satisfy the nutritional needs of offspring [1]. While mammary gland development has been studied to understand this normal physiological process, these studies are also important for cancer researchers as several of the processes involved in generating a fully functional mammary gland such as proliferation, differentiation, epithelial-to-mesenchymal transition (EMT), migration, and invasion, are hijacked by cancer cells [2–5]. The impact of genes, hormones and growth factors on ductal morphogenesis and alveolar development have been extensively investigated, while studies on non-coding RNAs such as microRNAs are less well characterized.

MicroRNAs (miRNAs) are small, non-coding RNA molecules 19–22 nucleotides (nt) long [6, 7]. miRNAs are initially transcribed as long primary transcripts that are processed by the ribonucleases Drosha [8, 9] and Dicer [8, 10, 11] into their mature 19–22nt duplexes [8, 12]. Mature miRNAs are incorporated into an RNA-induced silencing complex (RISC) [8] where they bind to the 3’-UTRs of mRNAs primarily using the miRNA seed region (nucleotides 2–8 of the miRNA) [8, 10, 11, 13, 14]. RISC complex binding to target mRNAs typically induces mRNA destabilization and translational repression [8, 10, 11, 13]. Each miRNA is predicted to target tens, hundreds, or thousands of mRNAs [15].

The miR-200 family consists of five members, miR-200a, miR-200b, miR-200c, miR-141, and miR-429 [16]. miR-200c and miR-141 are located on chromosome 12 in humans and chromosome 6 in mice [17]. miR-200b, miR-200a, and miR-429 are located on chromosome 1 in humans and chromosome 4 in mice [17]. The miR-200 family can be further characterized by seed sequences that separate the members into two functional groups. miR-200b, miR-200c, and miR-429 exhibit an identical seed sequence: AAUACUG [17]. Likewise, miR-200a and miR-141 share the seed sequence: AACACUG. The seed sequences of the two functional groups only differ by a single nucleotide (underlined) [17].

One of the main functions of the miR-200 family is influencing EMT. EMT is a biological process that causes polarized epithelial cells which interact with the basement membrane to lose their inter-cellular adhesion and acquire a mesenchymal cell phenotype [18–21]. This new phenotype has enhanced migratory capacity, invasiveness, and resistance to apoptosis [19–21]. EMT occurs in normal physiological events such as embryogenesis [22, 23], branching morphogenesis [24–27], and involution [28]. However, EMT is also implicated in abnormal physiological events such as tumorigenesis and metastasis [25, 29–32].

miR-200 expression appears to be controlled primarily by transcription factors such as Zeb1 and Zeb2 that directly bind to the promoter regions of the miR-200 clusters and repress transcription [33–35]. The miR-200 family and EMT-inducing transcription factors exist in a reciprocal negative feedback loop [24]. As a result, increasing miR-200 levels reduces EMT-inducing transcription factors. DNA methylation and histone H3 methylation also reduce miR-200 expression [36–41]; thus, inhibitors of DNA methylation or histone methylation should increase miR-200 expression. It has also been shown that compounds such as cryptotanshinone, phthalates, dihydrotanshinone, alkylphenols, and retinoic acid affect miR-200 levels [42–44].

The miR-200 family has been extensively studied in mammary tumorigenesis, particularly the claudin-low breast cancer subtype by our lab [41, 45, 46] and others [47–60]. From this research, it has been postulated that miR-200s may serve as therapeutic targets for the treatment or prevention of breast cancer. If miR-200s are to be used as therapeutic targets or preventative agents, it is important to understand the consequences that altering miR-200s may have on mammary gland development. This paper will provide the current state of the literature regarding the function of miR-200s in mammary ductal development and alveologenesis. For each stage, the general physiology of the mammary gland will be outlined, as will the known role of the miR-200 family.

## Main text

### The miR-200 family in embryonic and pubertal ductal morphogenesis

While most mammary ductal development occurs postnatally, the mammary ductal network is initiated in the embryo during mid-gestation in mammals [27, 61, 62]. In rodents, bands of ectodermal cells form along the mammary lines around embryonic day 11 (E11), and early mammary buds develop by E12.5 [27, 63, 64]. These mammary buds will continue to develop into an ectodermal stalk that extends into the mammary fat pad by E15.5 [27]. Programmed cell death removes cells from the centre of this stalk, creating a lumen in the centre of the duct [27]. The bifurcation of the stalk generates the initial ductal tree consisting of 10–15 branches by parturition [61]. Embryonic ductal development is regulated by many growth factors and hormones, including fibroblast growth factors, Wnt proteins and Hedgehog proteins that, in turn, regulate *Gata3*, *Hox*, *Tbx3*, and *Gli3* transcription factors [25, 27]. After birth, mammary ductal development is considered quiescent relative to embryonic and pubertal stages as the ductal growth rate matches normal body growth [2, 65, 66]. In response to pubertal hormones such as estrogen and progesterone, multilayer structures known as terminal end buds (TEBs) develop on the leading edge of the mammary duct and drive ductal elongation and bifurcation [66–68]. TEBs are composed of progenitor cells that eventually give rise to a single layer of luminal epithelial cells surrounded by a basal layer, including myoepithelial cells [69]. By the end of puberty, TEBs are no longer observable and the branched epithelium occupies 60% of the mammary stroma, leaving room for further expansion during lactation [70].

**Fig. 1:**
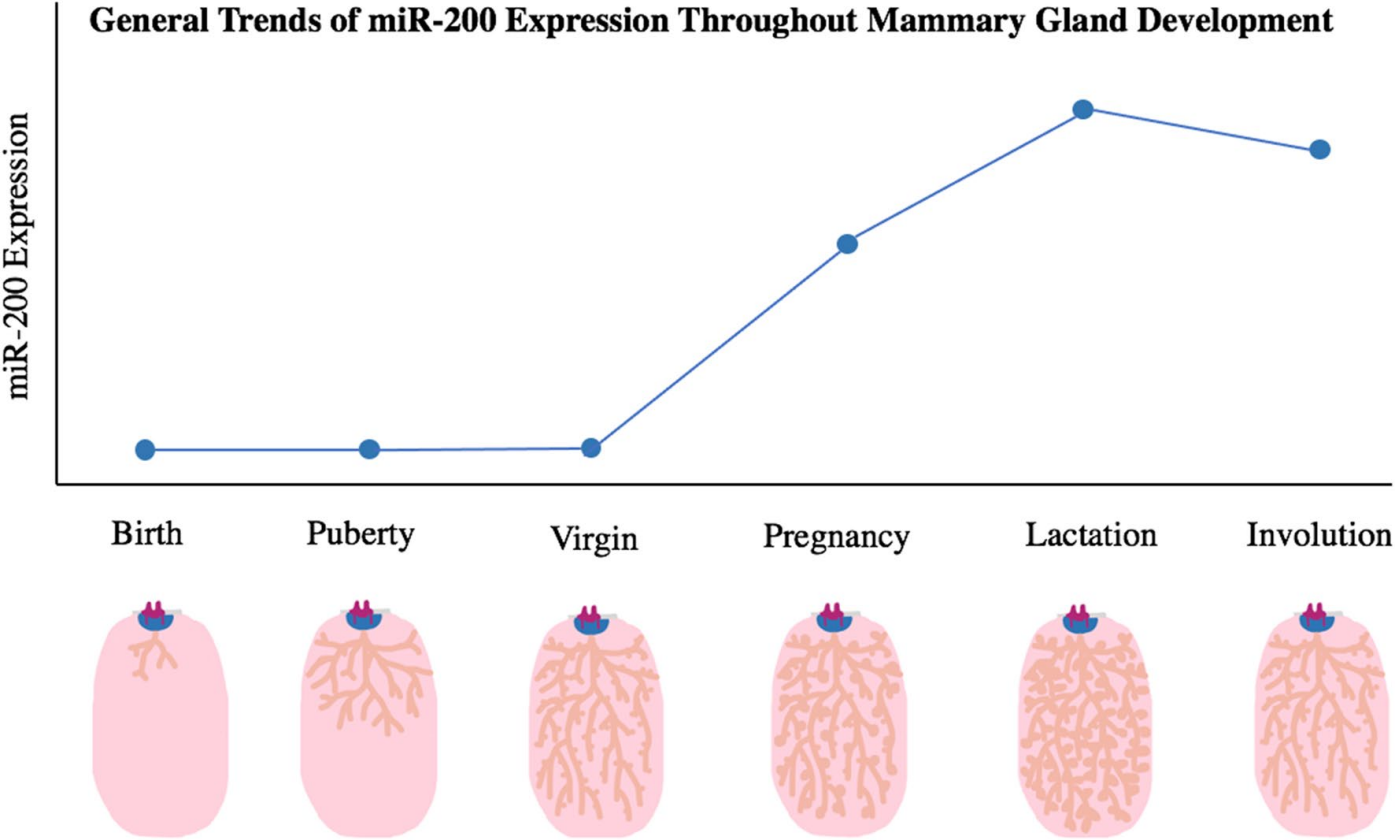
Illustrating the generalized trends of miR-200 expression throughout the main stages of mammary gland development. miR-200 expression is relatively low in the newborn, pubertal, and virgin mammary gland when EMT is a dominant process [71]. miR-200 expression increases throughout pregnancy, peaking during lactation and remaining high during involution to facilitate alveologenesis and support lactation [71, 75–78]. Mammary gland illustrations adapted from Macias & Hink [70]

While no studies have evaluated the role of miR-200s during embryonic mammary ductal morphogenesis, a limited number of studies have evaluated miR-200s in post-natal ductal development. Avril-Sassen et al. evaluated miRNA expression in whole murine mammary glands at different developmental stages and found miR-200a, miR-141, and miR-429 to be expressed at low levels in juvenile, pubertal, and mature virgin mammary glands (Fig. 1) [71]. The expression of miR-200b and miR-200c were not reported [71].

A study by Shimono et al*.* investigated mammary ductal development by transplanting 50,000 murine mammary epithelial cells infected with a miR-200c expressing lentivirus or a control lentivirus into the cleared fat pad of a syngeneic mouse [35]. While 11/20 transplants infected with the control lentivirus gave rise to a mammary ductal network, only 1/18 of the transplants infected with the miR-200c expressing lentivirus generated a mammary ductal tree [35]. Six of the miR-200c infected transplants produced small, disorganized clusters of cells. Staining of the transplants for myoepithelial cell marker keratin 14 (Krt14) and luminal cell marker keratin 18 (Krt18) revealed that the control infected transplants contain cells that were either Krt14 positive or Krt18 positive while the miR-200c infected transplants contained primarily Krt14 positive cells. This finding suggests that miR-200c promoted differentiation into myoepithelial cells rather than luminal cells.

The only study that evaluated miR-200s in human breast tissue was performed on reduction mammoplasty tissue. Bockmeyer et al*.* found that miR-200s are primarily expressed in luminal epithelial cells with lower levels of expression in myoepithelial cells [72].

Although these conclusions are based on a very small number of studies, it appears that miR-200s play a minor role in regulating ductal morphogenesis in virgin mice. Low levels of miR-200 family members throughout ductal morphogenesis [71] could indicate the presence of EMT-inducing transcription factors facilitating developmental changes requiring a mesenchymal cell phenotype [20]. Looking at this biological mechanism through the lens of breast cancer therapeutics, upregulating miR-200s in potential treatments could reduce necessary EMT-inducing transcription factors at this stage (ex. *Zeb1*, *Zeb2*). As Shimono et al*.* have begun to demonstrate, elevated expression of miR-200s may impair ductal elongation [35] and mammary trees favouring differentiation into myoepithelial cells over luminal cells would have later consequences during lactation. However, more research is required to determine whether elevated miR-200s delay or disrupt pubertal ductal morphogenesis and to define the mechanisms through which miR-200s impart this phenotype.

### The miR-200 family in alveologenesis

The first transformation seen in early pregnancy is an increase of ductal branching in preparation for alveolar development [70]. Proliferating epithelial cells produce alveolar buds which develop into alveoli [70]. Cellular differentiation becomes a dominant process at mid-pregnancy as cells prepare for lactation [73]. Hormonal stimulation to produce milk begins around day 16 of pregnancy in mice and is stimulated by estrogen, progesterone, and prolactin [74]. At the completion of lactation, weaning results in a lack of demand for breast milk causing the milk to stagnate within the epithelium. This initiates mammary involution, which remodels the extensive epithelial alveolar network back to its simple ductal structure exhibited before pregnancy [70].

Avril-Sassen et al*.* found that the expression of miR-200a, miR-141 and miR-429 were expressed at higher levels in whole murine mammary glands during pregnancy compared to virgin mammary glands and a further increase in miR-200a, miR-141 and miR-429 expression was observed in lactating mammary glands [71]. The levels of miR-200a, miR-141 and miR-429 remained highly expressed during involution [71].

Similarly, a study by Nagaoka et al*.* found that miR-200a expression increased in mid-pregnant murine mammary glands (gestation day 14) compared to virgin mammary glands and further increased in lactating mammary glands [75]. The increase in miR-200a expression in lactating mammary glands was associated with an increase in *Csn2* and *Cdh1* expression and a decrease in *Vim* expression [75]. Moreover, this group found that the normal mouse mammary epithelial cell line, EpH4, showed an increase in miR-200a expression as well as *Csn2* and *Cdh1* expression following treatment with a mixture of lactogenic hormones [75]. Knockdown of miR-200a in EpH4 cells prior to treatment with lactogenic hormones significantly decreased *Csn2* and *Cdh1* expression compared to control cells [75].

Galio et al. investigated mouse mammary tissue collected from virgin mice as well as mice during early pregnancy, late pregnancy, early lactation, and late lactation [76]. miR-200a, miR-200b, and miR-200c showed a similar expression profile with low levels of expression in virgin and early pregnancy mammary glands with increased expression in mammary glands during late pregnancy and lactation [76]. Mammary glands during late lactation had the highest expression of miR-200a, miR-200b, and miR-200c [76]. miR-141 and miR-429 expression were low in virgin and early gestation mammary glands and increased in late pregnancy and lactating mammary glands; however, the expression of miR-141 and miR-429 peaked in mammary glands during early lactation [76].

The increase in miR-200 expression during alveologenesis has also been observed in species other than mice. Galio et al*.* evaluated miRNA expression in cycling ovine mammary glands as well as ovine mammary glands during early, mid, and late pregnancy as well as during lactation [76]. miR-200a, miR-200b, miR-200c, and miR-141 were all shown to increase in expression in mammary glands during late gestation compared to the estrous cycling mammary gland with a further increase in lactating mammary glands [76]. Le Guillou et al*.* studied the mammary gland miRNA expression in bovines during lactation, revealing that miR-200a, miR-200b, miR-200c, and miR-141 were among the 30 most highly expressed microRNAs in mammary epithelial tissue [77]. Li et al*.* also found increased expression of miR-200c and miR-141 in lactating compared to non-lactating bovine mammary tissue [78]. Consistent with the increased miR-200 expression in mammary glands observed during lactation, miR-200 family members are also found at high levels in milk from humans [1, 79], mice [1], cows [1, 80], pigs [1, 81], and wallabies [1].

These studies consistently demonstrate that miR-200s increase in expression in mammary tissue during pregnancy and achieve peak expression during lactation. miR-200s, as well as other miRNAs, can be found in the milk of several species but whether there are any functional consequences of miR-200s in milk remains unknown (Fig. 1). One study found that miR-200c was found in bovine milk, but bovine miR-200c was not present in the circulation of individuals who drank bovine milk suggesting that milk miR-200s are unlikely to have significant physiological functions in newborns [82]. This finding by Auerbach et al*.* suggests that a miR-200-altering breast cancer treatment may not cause undue harm to the normal physiology of a newborn feeding from a mother with breast cancer. If future research can further support this hypothesis, safe breastfeeding throughout treatment would be a unique property of miR-200 therapeutics. Breast cancer patients receiving traditional chemotherapy and hormone therapies are discouraged from breastfeeding as anti-cancer medications (ex. Doxorubicin, Cisplatin, Mitoxantrone, Methotrexate) [83] and hormone receptor modulators (ex. Tamoxifen) [84] can be transferred to the infant through breastmilk with toxic effects. Surgical removal of breast tissue (lumpectomy or single mastectomy) under general anesthesia may also allow for safe breastfeeding [85, 86]; however, these treatment options may not be sufficient for aggressive subtypes of breast cancer. For an individual with breast cancer, a miR-200-altering therapy is unlikely to produce off-target effects on their normal mammary physiology throughout pregnancy and lactation as miR-200 levels are already high during the two developmental stages.

### miR-200 mRNA targets and mammary gland development

miR-200 family members exert their effects by binding to mRNA transcripts matching their seed sequence and repressing translation to prevent/reduce mRNA-specific protein synthesis [8, 10, 11, 13]. There are thousands of mRNAs targeted by miR-200s as Bracken et al*.* found 917 transcripts directly bound to miR-200a and 1,194 transcripts directly bound to miR-200b [87]. Among the miR-200 mRNA targets, *Ctnnb1*,* Zeb1*, *Zeb2*, *Snai1*, *Snai2*, and *Twist* will be discussed based on their importance throughout mammary gland development.

The finding by Shimono et al*.* that overexpressing miR-200c impaired the elongation of mammary ductal trees [35] can be understood by investigating miR-200 mRNA targets. Thus far, miR-200a [88, 89], miR-200b [90, 91], miR-200c [90, 92], and miR-141 [90] have been found to inhibit β-catenin (Ctnnb1) activation by targeting its mRNA. β-catenin inhibition prevents activation of transcription factor TCF which mediates the growth and proliferation outcomes of the Wnt signaling pathway [93]. Postnatally, Uyttendaele et al. found that Wnt-1 is involved in mammary branching morphogenesis by overcoming Notch-mediated inhibition [94]. For successful Wnt-mediated branching morphogenesis, miR-200 levels must be low which Avril-Sassen et al*.* confirmed for pubertal morphogenesis [71]. miR-200 members also bind to the mRNA of EMT-associated transcription factors *Zeb1* and *Zeb2* [34]. As epithelial cells invade the underlying matrix during pubertal ductal morphogenesis, it is hypothesized that cells undergo partial-EMT [26], and EMT-inducing transcription factors have been detected at TEBs during puberty [27]. This invasion would not be possible if cells remained in an epithelial cell phenotype promoted by miR-200s based on cell-adhesion to the basement membrane.

While stages leading up to pregnancy require low miR-200 expression, peaking miR-200s during and after pregnancy is essential for lactation and is facilitated by interactions with mRNA targets. An epithelial cell morphology within the lactiferous ducts is required during lactation as luminal cells must establish and maintain apical/basal polarity to function as secretory cells. Mesenchymal transcription factors *Zeb1*, *Zeb2*, [34, 95] *Snai1*, *Snai2,* and *Twist* [95] have been identified as direct targets of miR-200 family members based on their mRNA sequences. Watson et al*.* have also demonstrated miR-200s’ ability to significantly reduce the expression of *Zeb1*, *Snai1*, *Twist1*, and *Twist2* [45]. *Zeb1*, *Zeb2*, and *Snai1* proteins bind directly to the *Cdh1* promoter to repress its transcription while Twist proteins repress *Cdh1* indirectly [96]. Cdh1 is essential for lactation and Boussadia et al*.* demonstrated that *Cdh1* gene deletion impacted terminal differentiation of the lactating mammary gland. *Cdh1* gene deletion reduced milk production so significantly that adult mouse mothers could not suckle their offspring [74]. miR-200s translational repression of EMT-associated transcription factors through mRNA interaction prevents Cdh1n repression, contributing to successful lactation.

Based on the extensive number of mRNA targets by the miR-200 family, understanding their impact on the Wnt signaling pathway via β-catenin and EMT via *Zeb*, *Snai1*, *Snai2*, and *Twist* is just scratching the surface of understanding this microRNA family. However, these pathways serve as avenues for future research to further understand the impact of miR-200s on normal mammary gland development.

## Conclusions

During mammary ductal morphogenesis, where the percentage of progenitor cells is relatively high and cell migration and invasion are required for mammary ductal elongation, it appears that the expression of miR-200s is low. The results reported by Shimono et al*.* provide preliminary evidence that increased expression of one or more miR-200 family members can impair ductal elongation potentially by interacting with the Wnt/β-catenin signaling pathway and/or inhibiting EMT. However, supporting evidence is required to further substantiate these claims and confirm the biological mechanisms at play. Increased expression of miR-200 family members during a potential cancer therapy may consequently impair ductal morphogenesis. While breast cancer is rare in adolescents undergoing pubertal branching morphogenesis, it must be considered for post-pubertal individuals experiencing ductal morphogenesis associated with cyclic ovarian stimulation in preparation for lactation. Studies analyzing mammary duct length and area of miR-200 knockdown mice would provide valuable insights on the impact of miR-200-altering therapeutics on normal mammary development. Alveologenesis in late pregnancy and maintenance of alveoli structure and function during lactation are associated with increased expression of miR-200s. As mammary epithelial cells are terminally differentiated during alveolar development, the increase in miR-200s during this process is consistent with the observation that miR-200s are associated with maintaining an epithelial cell identity. While increased expression of miR-200s in alveoli is consistently observed, the requirement of miR-200 for proper alveolar development and the functions of miR-200s in alveolar epithelial cells have yet to be determined. Therefore, studies overexpressing or knocking down/knocking out miR-200s during different mammary developmental stages are required to determine the functional roles of miR-200s in the mammary gland. Future research should also focus on EMT-associated transcription factors such as *Zeb*, *Snai1*, *Snai2*, and *Twist* [33–35] to understand the complete picture of the miR-200 family throughout normal mammary development. Finally, studies providing insights into the mechanisms through which miR-200s impair mammary tumor initiation, growth and progression will help determine whether increased expression of miR-200s has the potential to serve as a therapeutic or preventative strategy for breast cancer.

## Data Availability

Not applicable.

## Abbreviations

Cdh1: E-cadherin
EMT: Epithelial-to-mesenchymal transition
Krt14: Keratin 14
Krt18: Keratin 18
miRNAs: MicroRNAs
RISC: RNA-induced silencing complex
TEB: Terminal end bud
